# YM155 exerts potent cytotoxic activity against quiescent (G_0_/G_1_) multiple myeloma and bortezomib resistant cells *via* inhibition of survivin and Mcl-1

**DOI:** 10.18632/oncotarget.22871

**Published:** 2017-12-04

**Authors:** Miyuki Ookura, Tatsuya Fujii, Hideki Yagi, Takuya Ogawa, Shinji Kishi, Naoko Hosono, Hiroko Shigemi, Takahiro Yamauchi, Takanori Ueda, Akira Yoshida

**Affiliations:** ^1^ Department of Hematology and Oncology, University of Fukui, Matsuoka, Fukui 910-1193, Japan; ^2^ Department of Pharmaceutical Sciences, International University of Health and Welfare, Otawara, Tochigi 324-8501, Japan; ^3^ Department of Hematology, International University of Health and Welfare Hospital, Iguchi, Nasushiobara, Tochigi, 329-2763, Japan

**Keywords:** YM155, survivin, Mcl-1, quiescent cells, multiple myeloma

## Abstract

YM155, a novel small molecule inhibitor of survivin, shows broad anticancer activity. Here, we have focused on the cytotoxic activity of YM155 against multiple myeloma (MM) including cytokinetically quiescent (G_0_/G_1_) cells and bortezomib resistant cells. YM155 strongly inhibited the growth of MM cell lines with the IC_50_ value of below 10 nM. YM155 also showed potent anti-myeloma activity in mouse xenograft model. YM155 suppressed the expression of survivin and rapidly directed Mcl-1 protein for proteasome degradation. YM155 abrogated the interleukin-6-induced STAT3 phosphorylation, subsequently blocked Mcl-1 expression and induced apoptosis in MM cells. Triple-color flow cytometric analysis revealed that YM155 potently induced cell death of MM cells in G_0_ phase. Quiescent primary MM cells were also sensitive to YM155. We established bortezomib-resistant MM cell line, U266/BTZR1, which possess a point mutation G322A. YM155 exhibited similar cytotoxic potency against U266/BTZR1 compared with parental cells. Interestingly, survivin expression was markedly elevated in U266/BTZR1 cells. Treatment with YM155 significantly down-regulated this increased survivin and Mcl-1 expression in U266/BTZR1 cells. In conclusion, our data indicate that YM155 exhibits potent cytotoxicity against quiescent (G_0_/G_1_) MM cells and bortezomib-resistant cells. These unique features of YM155 may be beneficial for the development of new therapeutic strategies to eliminate quiescent MM cells and overcome bortezomib resistance.

## INTRODUCTION

Multiple myeloma (MM) is characterized by the abnormal growth of malignant plasma cells in the bone marrow. Recently, the introduction of new drugs such as bortezomib and lenalidomide for the treatment of MM patients has significantly improved the clinical outcome [1, 2]. However, most of the patients relapse due to acquired drug resistance or expansion of residual clones that shows intrinsic resistance. More effective drugs are urgently required to overcome drug resistance. MM is a low growth fraction tumor with only a small percentage of myeloma cells in the S phase of the cell cycle [3, 4]. The majority of myeloma cells are quiescent. MM stem cells display relative quiescence, similar to other cancer stem cell (CSC) populations [5, 6]. Quiescent cells are generally insensitive to conventional anticancer agents [7–9]. Therefore, it is critical to search the drug, which efficiently kills the quiescent MM cells.

Survivin is a member of the inhibitor of apoptosis protein (IAP) family [10]. It works as an inhibitor of apoptosis and also acts as a critical molecule for cell division [10, 11]. Survivin is highly overexpressed in many malignancies [10] [12]. Growing evidences suggest that high levels of survivin expression may be involved in drug resistance in tumor [13, 14]. Overexpression of survivin in myeloma has been documented. A significant correlation between survivin expression and clinical outcomes of MM has been reported [15, 16]. In addition, survivin knockdown by RNA interference induces apoptosis in MM cells [15]. Recent studies indicate that survivin is also overexpressed in CSC and may contribute to survival and self-renewal of CSC [17–19]. Thus, targeting of survivin is an intriguing therapeutic strategy for MM. Indeed, several attempts have been made for the development of survivin inhibitor [20–22].

YM155 (sepantronium bromide), a novel small molecule inhibitor of survivin was identified by cell-based survivin gene promoter luciferase assay among chemical libraries [20]. YM155 displays broad antitumor activity *in vitro* [20, 23–26]. YM155 have shown its safety and tolerability in early phase clinical trials for a variety of human malignancies [27–31]. YM155 has been believed to exhibit its cell killing effect by the reduction of survivin expression in tumor cells. However, Glaros et al. found that YM155 eradicates tumor cells primarily by inducing DNA damage, not by survivin inhibition directly [32]. Thus, the precise mechanism of action of YM155 is not yet fully understood.

Here, we investigated the efficacy and mechanism of action of YM155 in human MM cells including bortezomib-resistant and quiescent cells. We found that YM155 exhibits robust cytotoxic activity in MM cells through downregulation of survivin and Mcl-1 protein. We have focused on the cell killing activity of YM155 against quiescent (G_0_/G_1_) MM cells. Three-color flow cytometric analysis showed that YM155 potently induced cell death in G_0_ phase MM cells. We also examined whether YM155 could exert cytotoxic activity against bortezomib-resistant MM cells (U266/BTZ1R), which shows survivin overexpression and possess a point mutation G322A. YM155 showed similar cytotoxic potency against U266/BTZR1 compared with parental cells. This is the first report to demonstrate that YM155 exhibits potent cytotoxic activity against MM cells in G_0_ phase and acquired bortezomib resistant cells. These unique features of YM155 may be beneficial for the development of effective therapeutic strategies to eliminate quiescent MM cells and overcome acquired bortezomib resistance.

## RESULTS

### YM155 potently inhibits the cell growth and induces apopvtosis in MM cells

**Figure 1:**
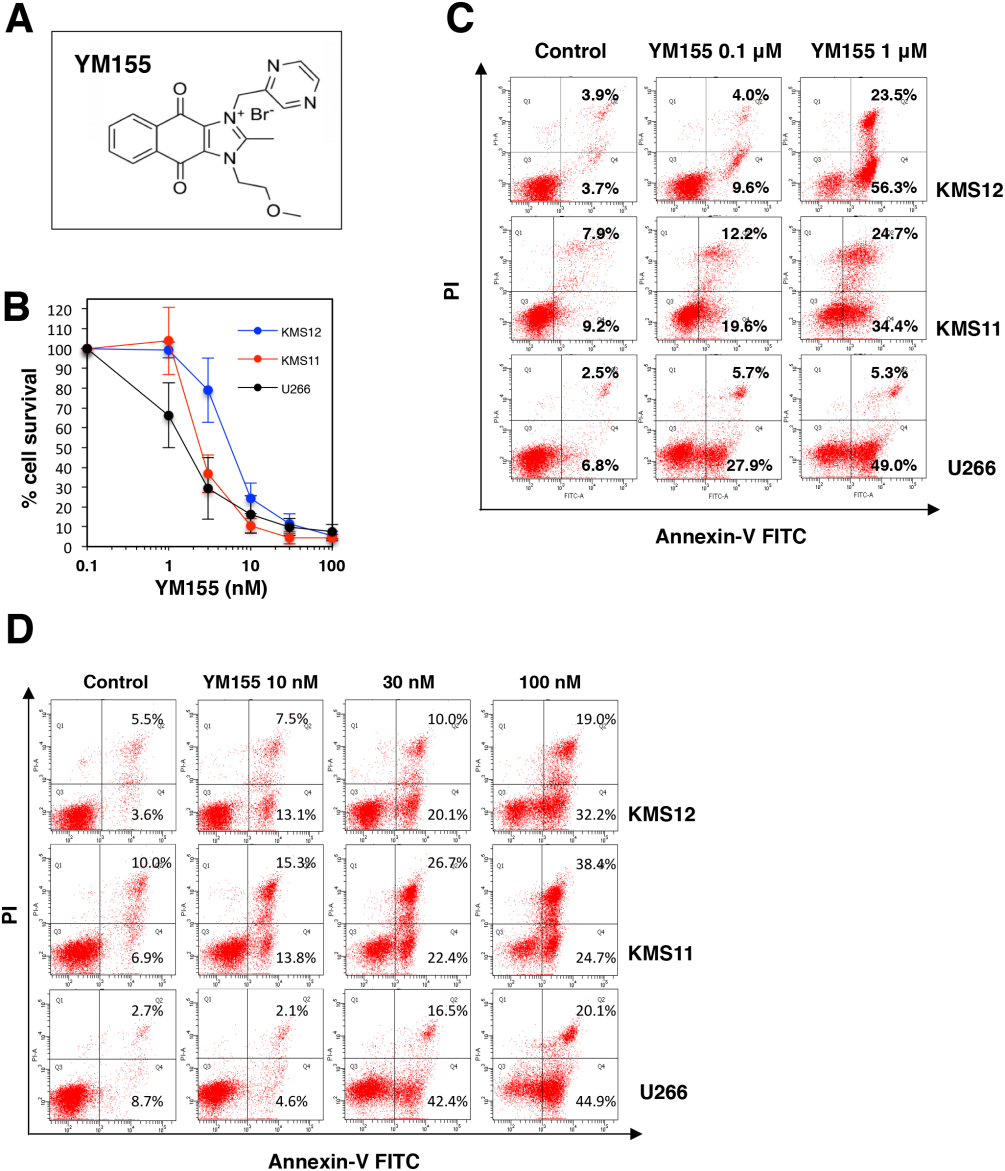
YM155 inhibits cell growth and induces apoptosis in MM cells **(A)** Chemical structure of YM155. Chemical name, 1-(2-methoxyethyl)-2-methyl-3-(pyrazin-2-ylmethyl) benzo[f]benzimidazol-3-ium-4,9-dione; chloride. **(B)** Cell growth inhibition by YM155 in human MM cell lines. The cells (U266, KMS11, and KMS12) were incubated with various concentrations of YM155 at 37°c for 72 h. Cell growth inhibition rate was determined by Cell counting Kit as described in Materials and Methods. IC_50_ was calculated as the mean of three independent experiments with triplicate determinations at each concentration. Error bars represent SD from triplicate experiments. **(C)** The cells (U266, KMS11, and KMS12) were incubated with 0.1 or 1 μM YM155 for 12 hr followed by Annexin-V and propidium iodide co-staining. **(D)** The cells (U266, KMS11, and KMS12) were incubated with 10, 30 or 100 nM YM155 for 24 h followed by Annexin-V and propidium iodide co-staining. The left lower quadrant shows live cells, the left upper quadrant necrotic cells, the right lower and the right upper quadrant early and late apoptotic cells, respectively. The percentage of cells in each quadrant is indicated.

The chemical structure of YM155 (Sepantronium bromide) is shown in Figure 1A. The anti-proliferative activity of YM155 against human myeloma cell lines KMS12, KMS11 and U266 cells was examined. Cells were treated with increasing concentrations of YM155 for 72 h, and then cell viability was measured by the cell counting kit-8. As shown in Figure 1B and Supplementary Figure 2, YM155 inhibited the cell growth of these cells in a dose dependent manner. The IC_50_ values of YM155 were 6.7 nM, 2.6 nM, and 1.9 nM for KMS12, KMS11 and U266 cells respectively. We investigated the effects of YM155 on the induction of apoptosis. MM cells were treated with various concentrations of YM155 and analyzed for induction of apoptosis using Annexin-V staining. YM155 increased cell populations in early and late stage apoptosis in dose dependent manner (Figure 1C and 1D).

### YM155 suppresses the expression of survivin in MM cells

**Figure 2:**
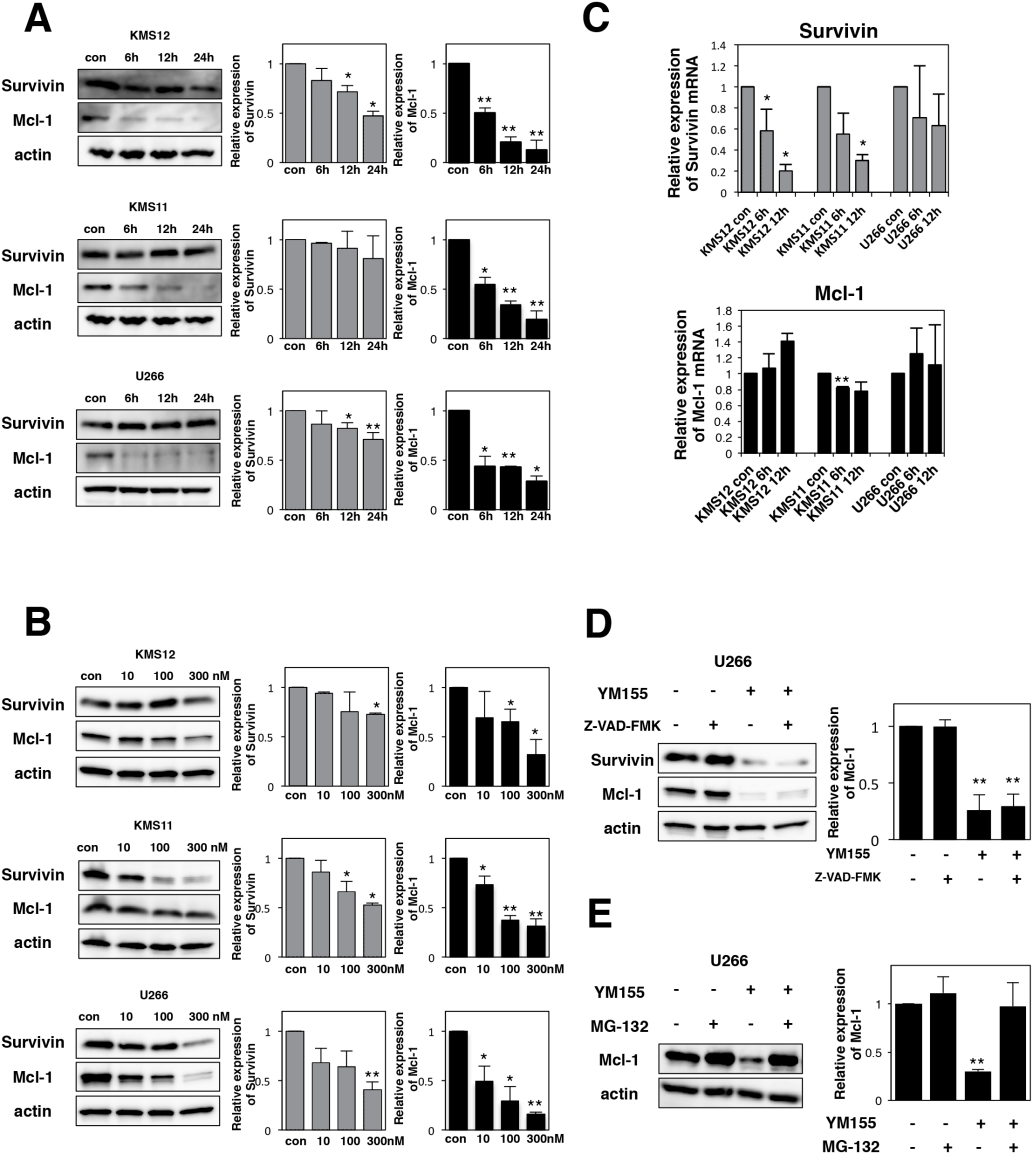
YM155 suppresses the expression of survivin and Mcl-1 protein **(A)** The cells (KMS12, KMS11, and U266) were treated with 0.1 μM YM155 for indicated time periods (control: 0.1% DMSO). **(B)** The cells were treated with 10, 100, or 300 nM YM155 for 24 hr. Expression levels of survivin and Mcl-1 protein were analyzed by immunoblotting (left panel) as described in Materials and Methods. Histograms (right panels) represent the ratio of band intensity of drug-treated to vehicle-treated, each normalized to background. Error bars represent SD from triplicate experiments. **(C)** Expression levels of survivin and Mcl-1 mRNA were analyzed by real-time RT-PCR analysis using 18s rRNA as an internal control. The cells (KMS12, KMS11, and U266) were treated with 1 μM YM155 for indicated time periods (control: 0.1% DMSO). After drug treatment, total RNA was prepared. Colums, mean from three separate experiments. ^*^P<0.05, ^**^P<0.01. **(D)** The U266 cells were incubated with 0.1 μM YM155 in the presence or absence of 30 μM Z-VAD-FMK for 24 hr. Z-VAD-FMK was added 60 min before addition of YM155. Survivin and Mcl-1 protein expression was analyzed by immunoblotting (left panel). Histograms (right panels) represent the ratio of band intensity of drug-treated to vehicle-treated, each normalized to background. Error bars represent SD from triplicate experiments. **(E)** The U266 cells were incubated with 0.1 μM YM155 or 20μM MG-132, alone or in combinations for 6 hours. MG-132 was added 60 min before addition of YM155. Mcl-1 protein expression was analyzed by immunoblotting (left panel). Histograms (right panels) represent the ratio of band intensity of drug-treated to vehicle-treated, each normalized to background. Error bars represent SD from triplicate experiments. ^**^P<0.01.

Using anti-survivin antibody, we conducted Western blotting analysis. YM155 decreased the levels of survivin protein in KMS12, KMS11 and U266 cells in a time- and dose-dependent manner (Figure 2A and 2B). The effects of YM155 on survivin mRNA expression were tested in MM cell lines by real time PCR analysis. The level of survivin mRNA was significantly suppressed by 1 μM YM155 treatment in KMS11 and KMS12 cells (Figure 2C).

### YM155 suppresses the expression of Mcl-1 via proteosomal degradation in MM cells

Mcl-1 is essential for the survival of MM cells [33, 34]. Our previous study reported that YM155 abolishes the expression of Mcl-1 in leukemia cells [35]. Therefore, the effects of YM155 on Mcl-1 protein expression were examined in MM cells by Western Blotting. The expression of Mcl-1 protein was profoundly suppressed by 6h YM155 treatment in all three myeloma cells in dose- and time-dependent manner (Figure 2A and 2B). Next, the expression of Mcl-1 mRNA was measured by qRT-PCR. We observed no obvious inhibitory effect of YM155 on the level of Mcl-1 mRNA in MM cells (Figure 2C). This result indicates that downregulation of Mcl-1 protein may be due to the transcription-independent mechanism. We considered the possibility that caspase-mediated degradation of Mcl-1 protein may be induced during apoptosis following YM155 treatment. However, Z-VAD-FMK, a pan-caspase inhibitor, did not block YM155 induced downregulation of Mcl-1 in U266 cells (Figure 2D). Next, we hypothesized that proteasome-mediated degradation of Mcl-1 may occur in MM cells after YM155 treatment. Western blot analysis revealed that proteasome inhibitor MG-132 significantly reversed the YM155-induced downregulation of Mcl-1 protein expression in U266 cells (Figure 2E), suggesting that YM155 promotes Mcl-1 degradation via activation of proteasome system.

### Effect of ectopic expression of survivin or Mcl-1 on YM155-induced cell death

**Figure 3:**
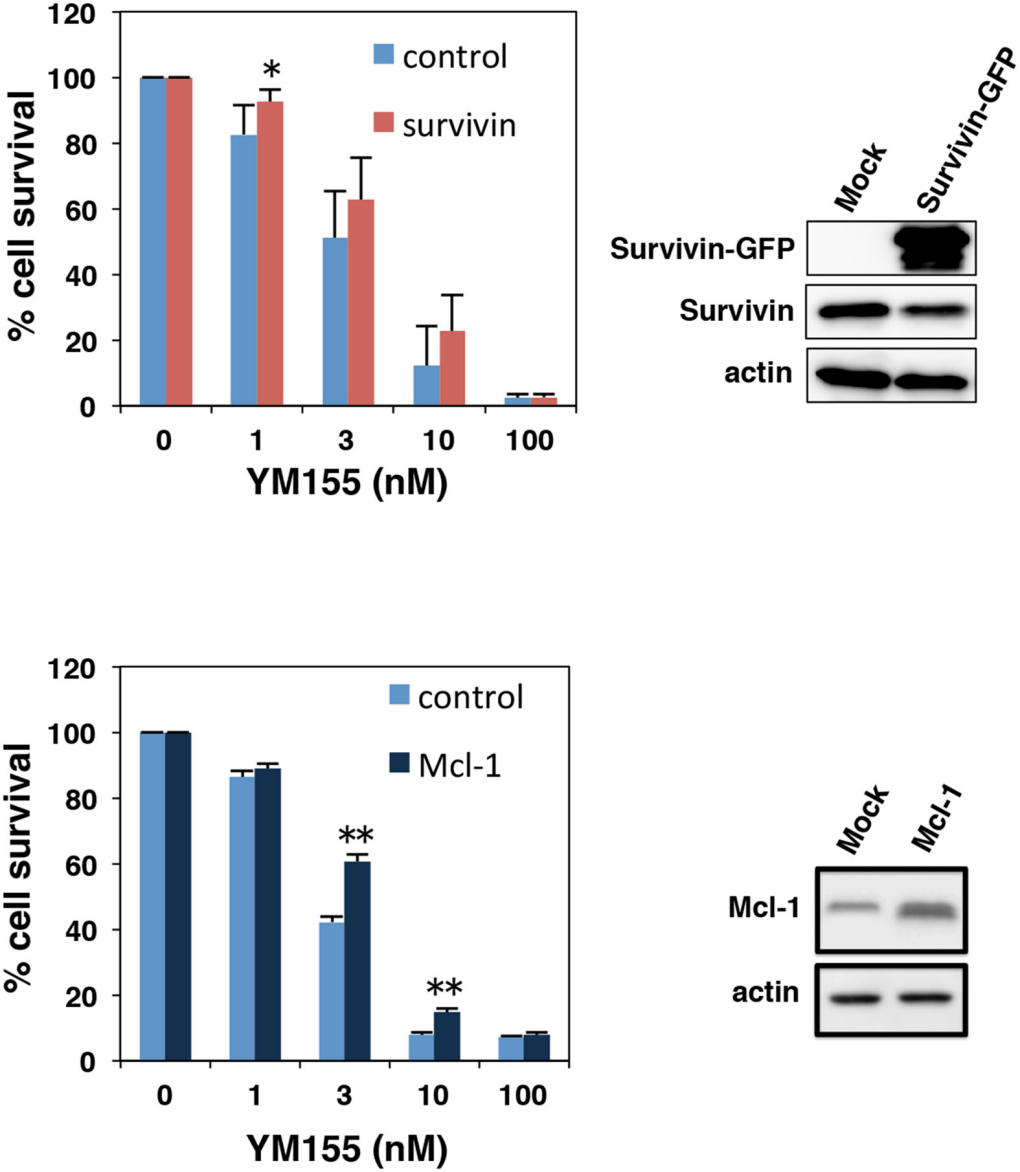
Effect of ectopic expression of survivin or Mcl-1 on YM155-induced cell death U266 cells were transduced with survivin-GFP or Mcl-1 retrovirus constructs. Ectopic expressions of survivin or Mcl-1 were confirmed by immunoblotting (right panels). The cells were incubated with various concentrations of YM155 at 37°c for 72 h. Cell growth inhibition rate was determined by trypan blue dye exclusion assay. All quantitative data are shown as the mean of three independent experiments. Error bars represent SD from triplicate experiments.

We conducted experiments to examine the effects of Mcl-1 or survivin overexpression on the cytotoxicity of YM155 in myeloma cells. U266 cells were transduced with survivin-GFP retrovirus or Mcl-1 retrovirus. As shown in Figure 3, both anti-apoptotic molecules showed protective effect against YM155 treatment. However, protective effect of Mcl-1 was more statically significant than that of survivin (Figure 3).

### YM155 blocks IL-6 induced signaling pathways in MM cells

**Figure 4:**
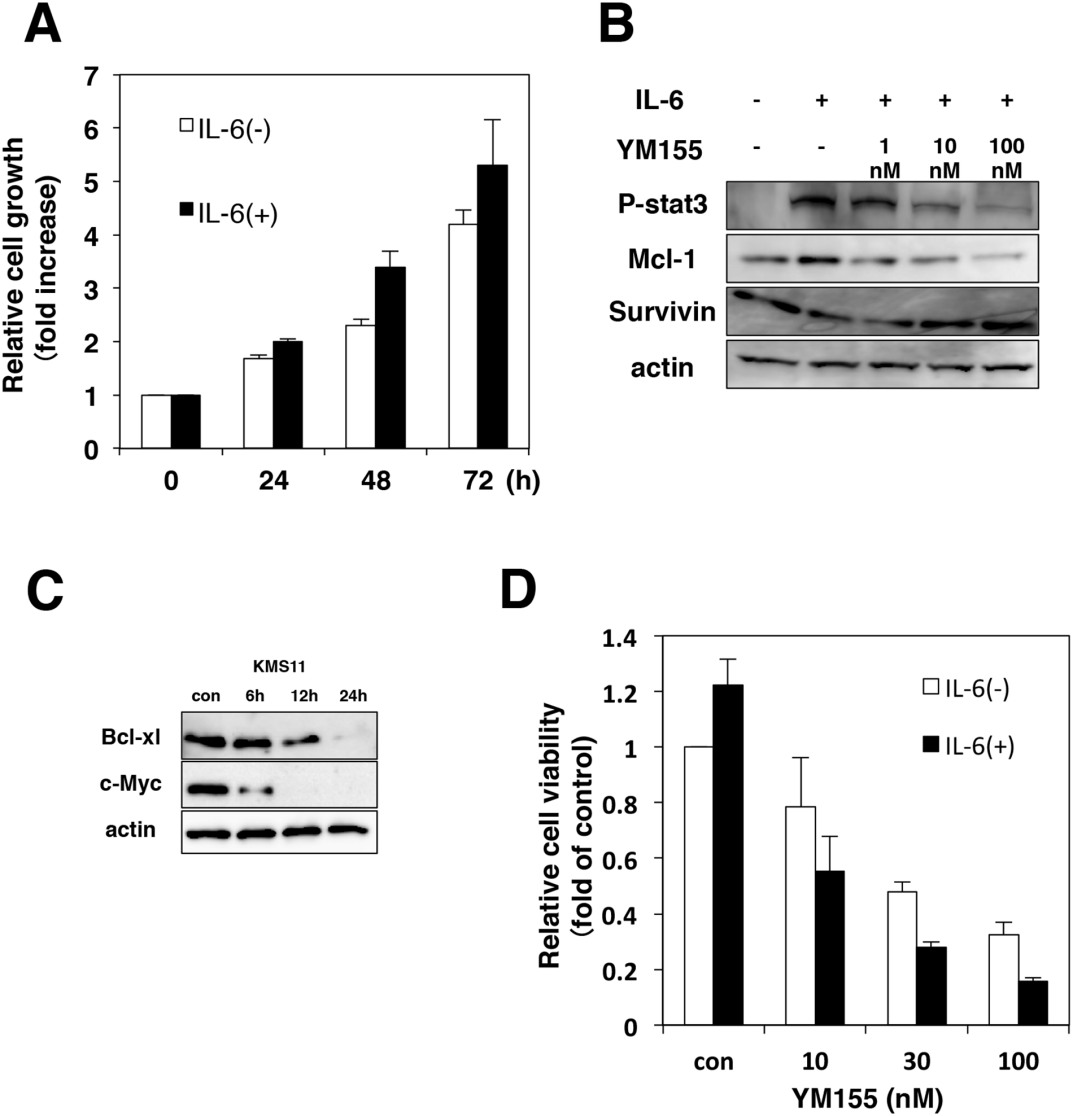
YM155 inhibits IL-6/STAT-3 signaling pathway **(A)** KMS12 cells were incubated with or without IL-6 (100 ng/ml) for indicated time periods. The cell growth was determined by trypan blue dye exclusion assay. All quantitative data are shown as the mean of three independent experiments. Error bars represent SD from triplicate experiments. **(B)** KMS12 cells were incubated with IL-6 (100 ng/ml) for 9 h in the presence or absence of YM155. YM155 was added 60 min after addition of IL-6. The phosphorylation of STAT3 and the expression of Mcl-1 and survivin protein were analyzed by immunoblotting analysis as described in Materials and Methods. **(C)** The expression levels of Bcl-xl and c-Myc protein were analyzed by immunoblotting. KMS11 cells were treated with 0.1 μM YM155 for indicated time periods (control: 0.1% DMSO). **(D)** KMS12 cells were incubated with YM155 at indicated concentrations in the absence or presence of IL-6 (100 ng/ml) for 48 hr. YM155 was added 60 min after addition of IL-6. The cell viability was determined by trypan blue dye exclusion assay. All quantitative data are shown as the mean of three independent experiments. Error bars represent SD from triplicate experiments.

Interleukin-6 (IL-6) is an essential growth factor for myeloma cells [2, 36]. Signal transducer and activator of transcription3 (STAT3) is a critical mediator of IL-6 signaling in MM cells [37, 38]. We examined the effect of IL-6 on cell growth in KMS12 cells. Cell growth of KMS12 was stimulated by the addition of IL-6 (Figure 4A). As shown in Figure 4B, when KMS12 cells were exposed to IL-6, phosphorylation of STAT3 was detected. Mcl-1 has been known to be the target gene of STAT3 [37]. Treatment of KMS12 cells with IL-6 increased the levels of Mcl-1 protein (Figure 4B). Treatment of KMS12 with YM155 inhibited IL-6 induced STAT3 phosphorylation and Mcl-1 upregulation in KMS12 cells (Figure 4B). Bcl-xL and c-Myc, have been known to be regulated by STAT3. YM155 treatment suppressed Bcl-xL and c-Myc protein expression in a time dependent manner (Figure 4C). Next, we examined whether IL-6 attenuates the cytotoxicity of YM155 against MM cells. As shown in Figure 4D, addition of IL-6 enhanced the cytotoxicity of YM155 in KMS12 cells.

### YM155 potently induces cell death in G_0_ MM cells

**Figure 5:**
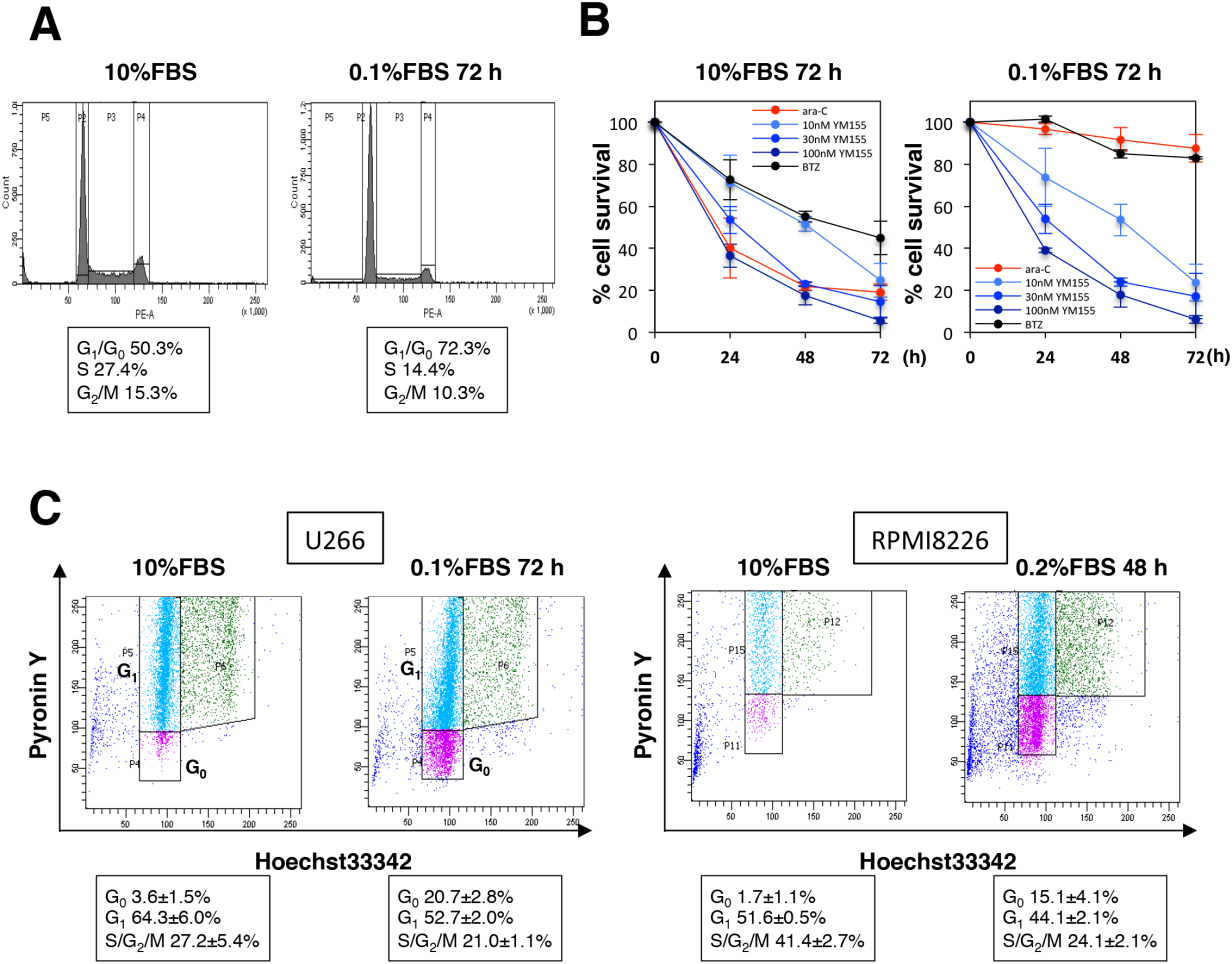

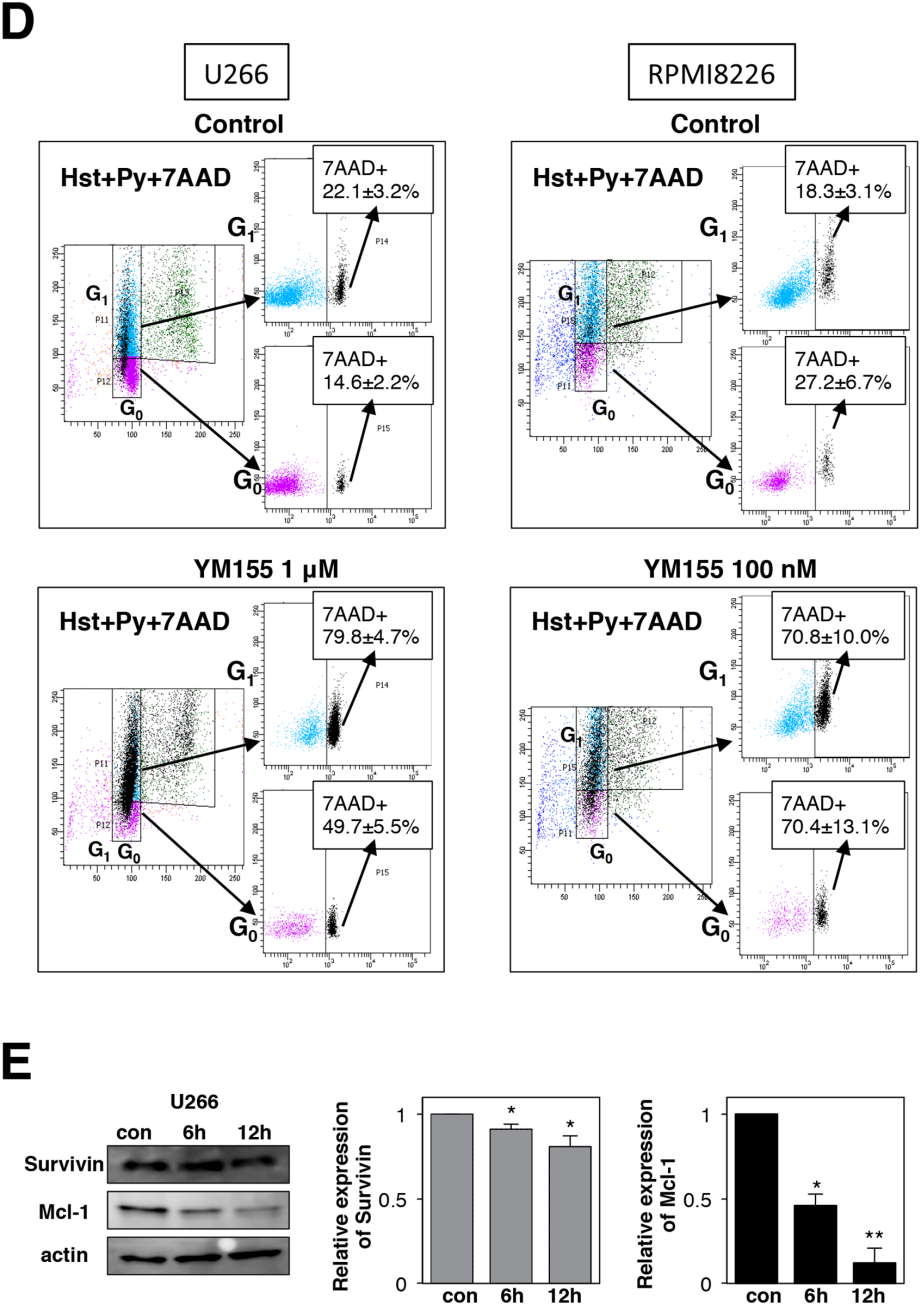
YM155 potently induces cell death in quiescent (G0/G1) MM cells **(A)** U266 cells were incubated in 0.1% or 10% FBS medium for 72 h, and cell-cycle profiles were examined by flow cytometry. **(B)** U266 cells were cultured in 0.1% or 10% FBS medium for 72 h, then the cells were incubated with various concentrations of YM155, 0.1 mM ara-C or 3 nM bortezomib at 37°c for indicated time periods. Cell survival inhibition rate was determined by trypan blue dye exclusion assay. **(C)** U266 cells were cultured in 0.1% or 10% FBS medium for 72h. RPMI8226 cells were cultured in 0.2% or 10% FBS medium for 48h. Flow cytometry was done to investigate G_0_ populations by double staining DNA with Hoechst33342 (Hst) and RNA with pyronin Y (PY). The G_0_ population (2N DNA/low levels of RNA, Hst+/PY-) was discriminated from the G_1_ population (2N DNA/high levels of RNA, Hst+/PY+). Results are representative of 3 separate sets of experiments. **(D)** G_0_-riched U266 or RPMI8226 cells were cultured in presence or absence (control: 0.1% DMSO) of YM155 (1 μM or 100 nM) for 24 hr. Triple color flow cytometeric analysis was performed to assess cell death (7-AAD positive cells) in G_0_ and G_1_ populations compared with control. Results (7-AAD positive cells) are representative of 3 separate sets of experiments. **(E)** U266 cells were cultured in low-serum-containing medium for 72 h, then the cells were incubated with 0.1 μM YM155 at 37°c for indicated time periods. The expression of survivin and Mcl-1 protein was analyzed by immunoblotting analysis. Histograms (right panels) represent the ratio of band intensity of drug-treated to vehicle-treated, each normalized to background.

In order to examine if YM155 exhibits cell killing activity in quiescent (G_0_/G_1_) MM cells, cells were cultured in low-serum medium to enrich the G_0_/G_1_ population. U266 cells incubated in 0.1% FBS medium exhibited no growth with approximately 70% of cells enriched in G_0_/G_1_ phase (Figure 5A), but there were almost no differences in cell viability. Notably, G_0_/G_1_-enriched cells were equally sensitive to YM155 compared with controls cultured in 10% FBS (Figure 5B). In contrast, the S-phase specific anticancer agent, ara-C did not show cytotoxic activity against MM cells cultured in low-serum medium (Figure 5B). Proteasome inhibitor bortezomib showed weak cytotoxicity in low-serum medium (Figure 5B). In order to discriminate G_0_ cells from G_1_ population, we employed a multiparameter flow cytometric analysis, which can identify G_0_ cells showing Hoechst33342 (Hst) positivity (Hst+, 2N diploid cells) but lower pyronin Y (PY) uptake (PY-, very low level of RNA synthesis in G_0_) from G1 (Hst+/PY+). As shown in Figure 5C, flow cytometric analysis showed that U266 cells cultured in low-serum medium displayed an enriched Hst+/PY- (G_0_) population, compared with 10% FBS controls (20.7±2.8% vs. 3.6±1.5%). 7-AAD staining was used to detect cell death in Hst+/PY- (G_0_) population. After 24 h of treatment with YM155, the Hst+/PY- (G_0_) population showed increase in 7-AAD uptakes over untreated controls (49.7±5.5% vs. 14.6±2.2%) (Figure 5D). YM155 also induced massive cell death of U266 in G_1_ phase (79.8±4.7% vs. 22.1±3.2%) (Figure 5D). We performed similar experiment in RPMI8226 cells. As shown in Figure 5C and 5D, RPMI8226 cells enriched in G_0_ phase were obtained by culturing in 0.2% FBS medium for 48 hr. Increased Hst+/PY- (G_0_) population was observed, compared with 10% FBS controls (15.1±4.1% vs. 1.7±1.1%) (Figure 4C). YM155 treatment induced cell death in the Hst+/PY- (G_0_) populations (70.4±13.1% vs. 27.2±6.7%) (Figure 5D). Significant increases in cell death of G1 phase also occurred (70.8±10.1% vs. 18.3±3.1%). These data indicated that YM155 can induce cell death in quiescent (G_0_ and G_1_) cells. In order to access the further insight of mechanism of cell killing activity of YM155, we tested the effect of YM155 on the expression of survivin and Mcl-1 in quiescent cells. Expression of Mcl-1 protein was more strongly inhibited by YM155 than that of survivin, although YM155 suppressed both survivin and Mcl-1 protein expression in quiescent U266 cells (Figure 5E).

### YM155 exhibits potent cytotoxicity in primary MM cells

**Figure 6:**
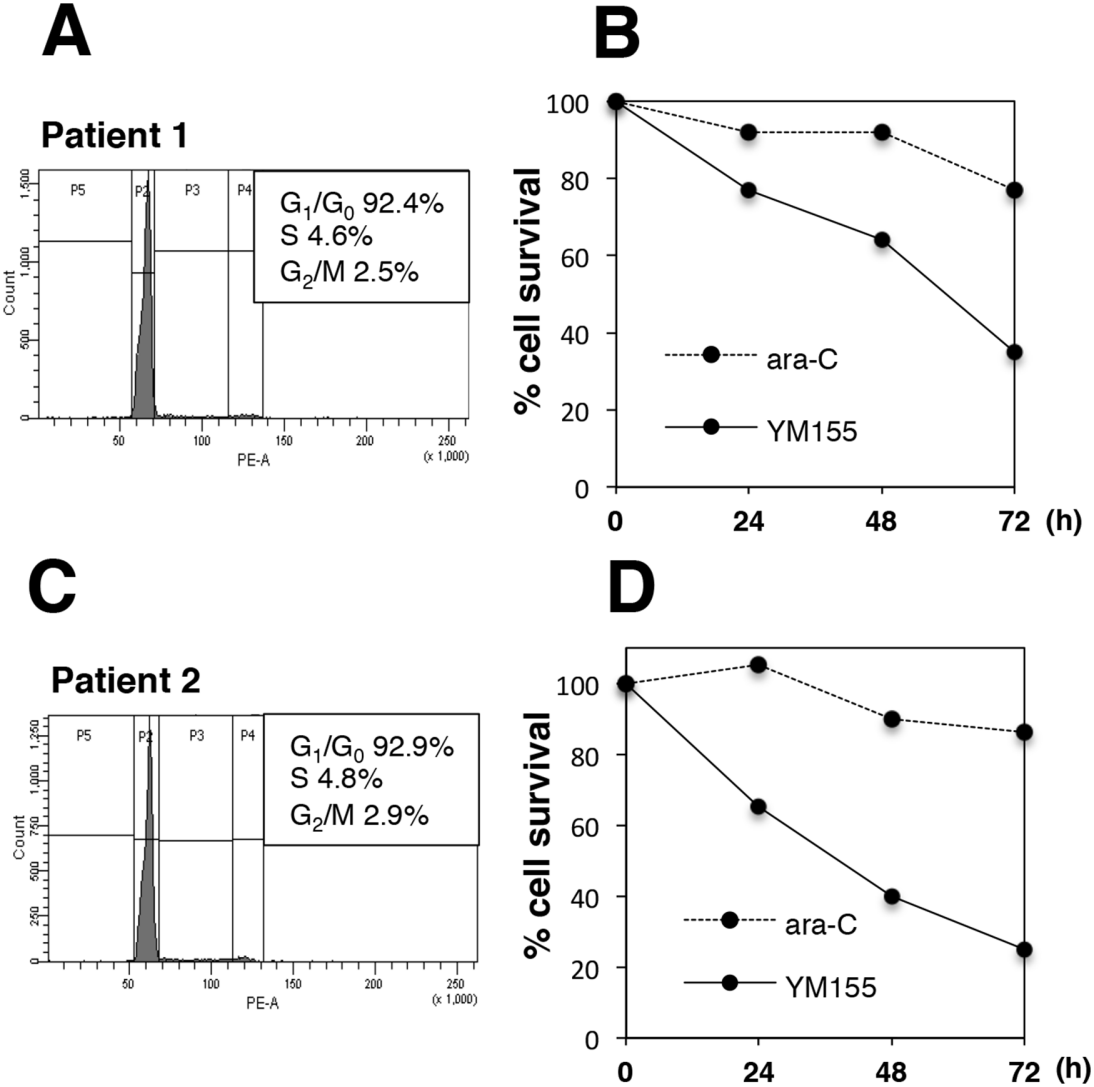
YM155 exerts potent cytotoxicity against primary MM cells Mononuclear cells were separated from bone marrow samples of two MM patients (patient No1 and No2) using ficoll-paque solution. Percentage of myeloma cells in mononuclear fraction was more than 80 %. The primary myeloma cells form patients No.1 **(A, B)** or No. 2 **(C, D)** were incubated with 100 nM YM155 or ara-C 0.1 mM at 37°c for indicated time periods. Cell-cycle profiles were analyzed by flow cytometry using PI staining. The cell survival was determined by trypan blue dye exclusion assay.

We tested if YM155 could show cell killing activity in primary MM cells. Flow cytometric analysis revealed that the majority of primary MM cells were in quiescent (G_0_/G_1_) phase (Figure 6A and 5C). YM155 exhibited potent cytotoxicity in primary MM cells (Figure 6B and 6D). On the other hand, S phase specific drug, ara-C did not show cytocidal effect cell in quiescent primary MM cells (Figure 6B and 6D).

### Anti-myeloma activity of YM155 *in vivo*

**Figure 7:**
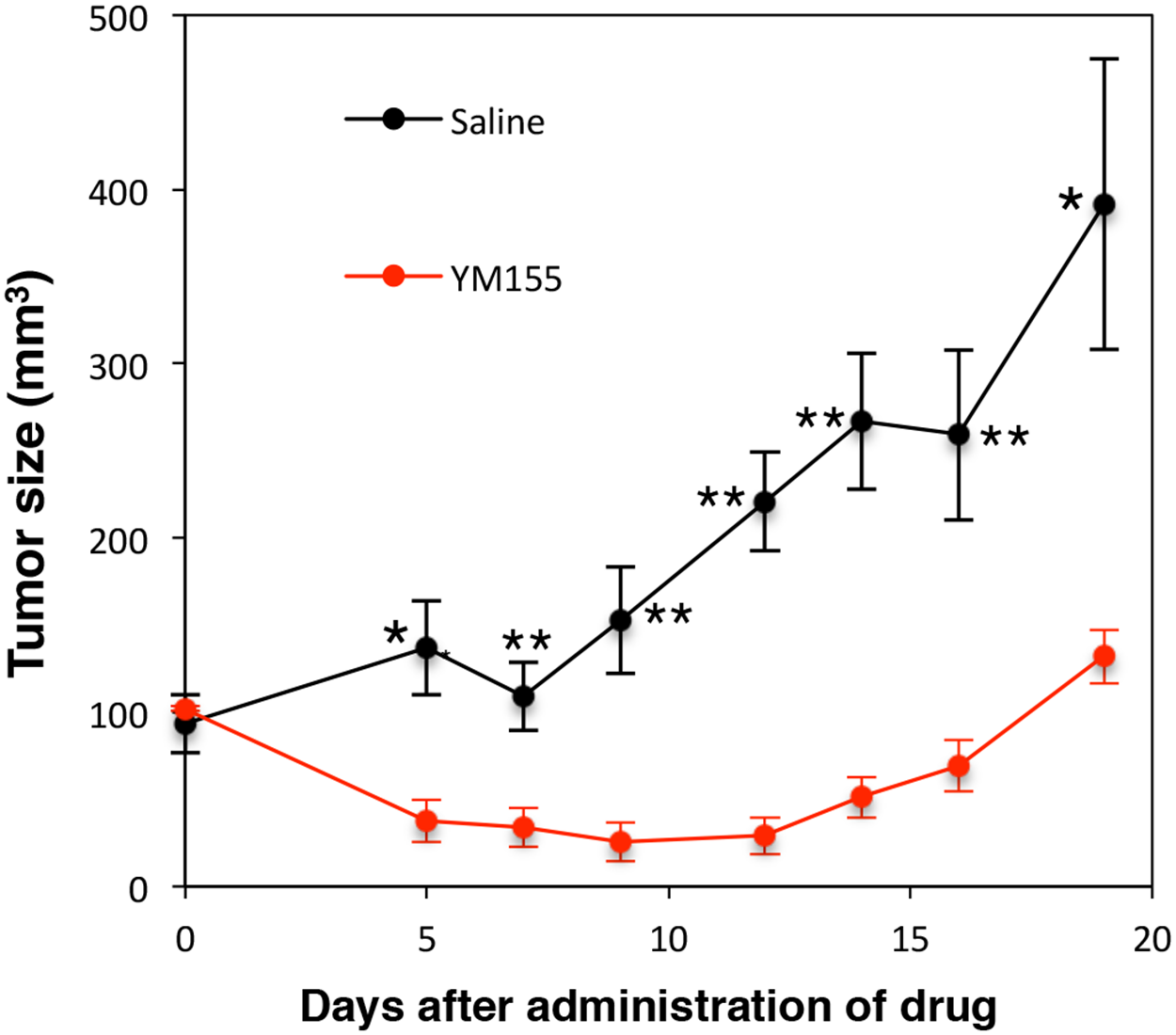
Anti-myeloma activity of YM155 *in vivo* NOD/SCID mice were inoculated subcutaneously with 1.7 × 10^7^ cells of RPMI8226 into the flank. Mice were treated with 5mg/kg of YM155 intraperitoneally once daily for 9 days (n = 6) or the vehicle (Control) on the same schedule (n = 6). Caliper measurements of the longest perpendicular tumor diameters were performed as described in materials and methods. ^*^P<0.05, ^**^P<0.01.

Next, we confirmed the cytotoxic activity of YM155 against MM cells *in vivo*. RPMI8226 cells were inoculated into NOD/SCID mice. YM155 was administered intraperitoneally once daily at 5mg/kg for 9 days. YM155 significantly inhibited the tumor growth in mice inoculated with RPMI8226 cells (Figure 7).

### YM155 overcomes bortezomib resistance in MM cells

**Figure 8:**
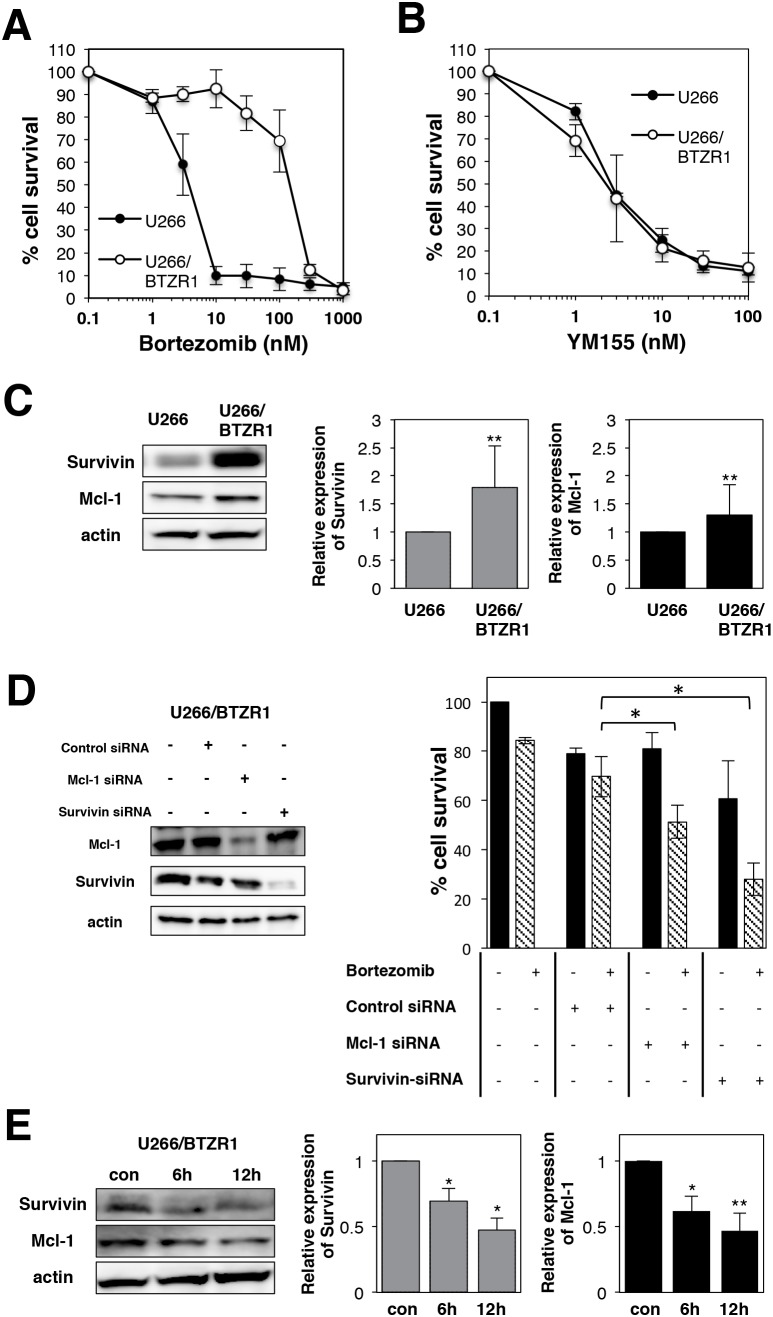
YM155 overcomes bortezomib resistance in MM cells **(A)** Cell growth inhibition by bortezomib in U266 or U266/BTZR1 cells. **(B)** Cell growth inhibition by YM155 in U266 or U266/BTZR1 cells. The cells were incubated with various concentrations of bortezomib (A) or YM155 (B) at 37°c for 72 h. Cell growth inhibition rate was determined by Cell counting Kit as described in Materials and Methods. IC_50_ was calculated as the mean of three independent experiments with triplicate determinations at each concentration. **(C)** Expression levels of survivin and Mcl-1 protein in U266 or U266/BTZR1 cells were analyzed by immunoblotting (left panel). Histograms (right panels) represent the ratio of band intensity compared to its parental cells, each normalized to background and band intensity of actin. Error bars represent standard deviation (SD, n ≥ 3). **(D)** U266/BTZR1 cells were transfected with siRNA against survivin or Mcl-1 by electroporation. Cells were also transfected with control siRNA. Proteins were extracted from the transfected cells 24 h after electroporation. Expression levels of survivin and Mcl-1 protein were analyzed by immunoblotting as described in Materials and Methods (left panel). Twenty-four hours after electroporation, U266 cells were incubated with 30 nM bortezomib for 72 h. The cell growth was determined by trypan blue dye exclusion assay. All quantitative data are shown as the mean of three independent experiments. Error bars represent SD from triplicate experiments (right panel). **(E)** Expression levels of survivin and Mcl-1 protein were analyzed in U266/BTZR1 cells incubated with YM155 by immunoblotting (left panel). The U266/BTZR1 cells were treated with 0.1 μM YM155 for indicated time periods (control: 0.1% DMSO). Histograms (right panels) represent the ratio of band intensity of drug-treated to vehicle-treated, each normalized to background. Error bars represent standard deviation (SD, n ≥ 3).

To examine the mechanism of bortezomib resistance, bortezomib-resistant MM cell lines U266/BTZR1 were established by continuous exposure to bortezomib. Resistant cell line was designated as U266/BTZR1. DNA sequencing analysis revealed that U266/BTZR1 cells have a typical point mutation, G322A in the exon2 of proteasome β5 subunit gene. This point mutation is an important mechanism of bortezomib resistance [39]. Cell growth inhibition assay showed 39-fold (IC50: 168 nM) levels of resistance in U266/BTZR1 compared with wild-type U266 cells (IC50: 4.3; Figure 8A). Interestingly, U266/BTZR1 cells showed a significant increase in the levels of Mcl-1 and survivin protein compared with parental U266 cells (Figure 8C). To examine the role of overexpressed Mcl-1 and survivin in bortezomib-resistance, U266/BTZR1 cells were transfected with siRNA against Mcl-1 or survivin. Western blotting analysis demonstrated that siRNA against Mcl-1 or survivin downregulated their protein expressions (Figure 8D). As shown in Figure 8D (right panel), combined treatment with bortezomib and Mcl-1 siRNA or survivin siRNA significantly inhibited the cell growth in comparison with bortezomib alone. We then tested the effects of YM155 on bortezomib-resistant MM cells. Treatment of U266/BTZR1 cells with YM155 potently suppressed this overexpressed survivin and Mcl-1 in a time dependent manner (Figure 8E). YM155 exhibited similar cytotoxic potency against U266/BTZR1 compared with its parental cells (Figure 8B), suggesting that YM155 can overcome bortezomib resistance.

## DISCUSSION

We demonstrated for the first time that YM155 exerts potent cytotoxic activity against MM cells in G_0_ and G_1_ phase population and bortezomib resistant cells via inhibition of survivin and Mcl-1. Our study unveils the novel and intriguing character of YM155. YM155 may have potential benefits for the treatment of MM.

The most challenging hurdle to the treatment of cancer is the resistance of quiescent cells (G0) including CSCs to conventional chemotherapeutic drugs. The majority of clinically utilized anticancer drugs are classical cytotoxic agents. Their common mechanism of action is preferential killing of dividing tumor cells by either inhibition of DNA and RNA synthesis or interruption of mitotic process. Proteasome inhibitor bortezomib effectively kills dividing myeloma cells. However, quiescent myeloma cells are relatively insensitive to bortezomib treatment [40]. This is one possible reason why myeloma patients eventually relapse following bortezomib based chemotherapy. Here, we found that, in addition to cycling cells, quiescent (G_0_/G_1_) MM cells are highly sensitive to YM155, whereas these cells were insensitive to ara-C treatment. We also found that YM155 was highly active against primary MM cells, which have a low proliferative capacity. In quiescent cells, we observed that YM155 significantly reduced the level of Mcl-1 protein as well as survivin in MM cells. Not only inhibition of survivin, but also suppression of Mcl-1 may be involved in the cell killing activity of YM155 in quiescent MM cells. Very recently, Kotschy et al. [41] successfully developed the potent small molecule Mcl-1 inhibitor of S63845. It is intriguing to consider the possibility that S63845 might possess the significant activity against quiescent MM cells.

Until now, there have been only a few studies, which describe the effective elimination strategies against quiescent (G_0_/G_1_) MM cells. Interestingly, Pei et al. [42] reported that coexposure to the checkpoint kinase-1 (Chk1) and MEK1/2 inhibitors AZD7762 and selumetinib (AZD6244) robustly induced apoptosis in quiescent (G_0_/G_1_) MM cells. The author suggested the possibility that intact Chk1 function may be required even in quiescent cells to keep the genomic stability by preventing the spontaneously occurring DNA damage. Schewe et al. [40] found that treatment with bortezomib eradicated the majority of replicating MM cells but invariably left behind a surviving cell fraction in quiescent (G_0_/G_1_) phase. Addition of the phosphatase inhibitor salubrinal can eliminate this quiescent MM cells [40].

In our study, a decrease in the level of Mcl-1 mRNA after YM155 treatment was not detected, although YM155 potently reduced the level of Mcl-1 protein in MM cells. We observed that treatment of U266 cells with proteasome inhibitor MG132 reversed YM155-induced downregulation of Mcl-1 protein expression, suggesting that YM155 promotes Mcl-1 degradation via activation of proteasome system. Potent reduction of the level of Mcl-1 protein by YM155 may play an important role in cell killing in MM cells. Mcl-1 has a critical role in MM cell survival and proliferation [33, 34]. These facts make Mcl-1 a potential therapeutic target for MM [43].

YM155 exerted potent activity against U266/BTZR1 cells, which showed approximately 39-fold levels of resistance to bortezomib. We also examined the cytotoxic effect of YM155 on the growth of another highly bortezomib resistant U266 cells, designated as U266/BTZR2. Cell growth inhibition assay showed approximately 100-fold levels of resistance in U266/BTZR2 compared with wild-type cells. One hundredfold resistance is too high from a clinical point of view. However, relatively low concentration of YM155 (30 nM) strongly inhibited the growth of U266/BTZR2 cells (Supplementary Figure 1).

In the literature, U266/BTZR1 is the first bortezomib resistant MM cell line, which overexpresses survivin. Survivin overexpression may be involved in the bortezomib resistance. Notably, YM155 exhibited similar cytotoxic potency in U266/BTZR1 compared with parental cells. Wagner et al. [16] reported that growth inhibition by YM155 was rescued by ectopic expression of Mcl-1 but not survivin, identifying Mcl-1 as a determinant of cytotoxicity of YM155 in MM cells. We observed that treatment with YM155 significantly reduced the levels of overexpressed survivin and Mcl-1 protein in U266/BTZR1 cells. In the present study, our data showed that silencing of Mcl-1 or survivin could re-sensitize U266/BTZR1 cells to bortezomib. Thus, it is interesting to consider the possibility that reduction of survivin protein by YM155 may reverse the bortezomib resistance. Pretreatment of bortezomib resistant cells with YM155 may enhance the cytotoxic efficacy of proteasome inhibitor.

Interleukin-6 (IL-6) is a critical growth factor for myeloma cells [36]. IL-6 upregulates Mcl-1 expression *via* activated STAT3 signaling in myeloma cells [37, 38]. Blocking this pathway is a potential therapeutic strategy for MM. Here, our data clearly showed that YM155 inhibited the phosphorylation of STAT3 following IL-6 stimulation, subsequently suppressed Mcl-1 expression and induced apoptosis. Abrogation of IL-6 induced STAT3 signaling network by YM155 may cause growth inhibition of MM cells even in presence of stromal cells.

Cytocidal action of YM155 has been attributed to the blocking of survivin gene promoter activity through inhibition of transcription factor such as ILF3 and Sp1 [44–46]. However, it has been reported that YM155 inhibits the expression of FGFR [47], XIAP [48] and AKT [49] protein. It seems that YM155 shows the unique inhibitory activities towards multiple critical proteins in tumor cells. These diverse effects of YM155 cannot be explained by its inhibitory action on survivin promoter. Further investigations will be required to clarify this point.

In conclusion, we found that YM155 exerts robust anti-myeloma activity through inhibition of survivin and Mcl-1. Furthermore, we demonstrated that YM155 exhibits substantial cytotoxicity against quiescent MM cells and acquired bortezomib- resistant cells. These unique properties of YM155 warrant further evaluation of this unique compound in clinical trials for patients with MM.

## MATERIALS AND METHODS

### Cell culture

The human MM cell line U266 and RPMI8226 were purchased from the American Type Culture Collection (ATCC) (Manassas, VA, USA). KMS11 and KMS12 were obtained from Japanese Collection of Research Bioresources Cell Bank (JCRB Cell Bank) (Osaka, Japan). Cells were cultured in RPMI 1640 supplemented with 10% FCS (Sigma, St. Louis, MO) at 37°c under 5% CO_2_ in a humidified atmosphere. Exponentially growing cells were exposed to drugs for the indicated time periods.

### Establishment of bortezomib-resistant cell line

Bortezomib-resistant U266 cell line was obtained by stepwise increasing concentrations of bortezomib over a period of 7 months, starting at a concentration of 2 nM bortezomib. Established bortezomib-resistant cell line was designated as U266/BTZR1.

### Chemicals and antibodies

YM155 was obtained from Selleck chemicals (Houston, TX). Ara-C was obtained from WAKO (Tokyo, Japan). Bortezomib (PS-341) was obtained from Takeda Pharmaceutical Company Limited (Osaka, Japan). IL-6 was obtained from Chugai Pharmaceutical (Tokyo, Japan). MG-132 was obtained from Abcam (Cambridge, UK). Z-VAD-FMK was obtained from MBL (Nagoya, Japan). The following antibodies were used: Survivin, STAT3, Mcl-1, Bcl-xL and c-Myc from Cell Signaling Technology (Danvers, MA); phospho- STAT3 from American Research Products (Waltham, MA); Actin from Sigma-Aldrich.

### Cell growth inhibition assay

Cells were continuously exposed to the drug for 72 hours in triplicate. Cell viability was assessed with the Cell counting kit-8 (CCK-8) assay (Dojindo, Japan). The absorbance was measured by plate reader (SPECTRA MAX 250, Molecular Devices) with 450 nm wavelength.

### Trypan blue exclusion assay

Cell viability was calculated by Trypan blue exclusion assay. Cell suspension was simply mixed with 0.5% Trypan blue solution (Nacalai tesque, Kyoto, Japan) and then visually examined to determine whether cells take up or exclude dye. Viable cells, which are unstained, appear clear. Non-viable cells appear dark blue colored. The number of dark blue stained cells and non-stained cells were counted on Burker-Turk hemocytometer through a microscope.

### Annexin-V binding assay

Apoptotic cells were detected after drug treatment using annexin V and propidium iodide (PI) co-staining (Annexin-V-FLUOS Staining Kit, Sigma, St. Louis, MO) coupled with flow cytometry (BD, FACSCanto II) according to the manufacturer's instructions.

### Quantitative real-time RT-PCR (qRT-PCR)

Total RNA was isolated from MM cells using RNeasy mini kit (QIAGEN) and analyzed by qRT-PCR using the StepOnePlus PCR system (Applied Biosystems). RNA was amplified with One-step RT-PCR master mix reagent kit and Taqman Gene Expression Assays (Applied Biosystems, CA): The assay IDs were: BIRC5 (Survivin), Hs03043574_m1; Mcl-1, Hs0150896_m1; GAPDH, Hs02786624_g1; 18s rRNA, Hs99999901_s1. The relative mRNA expression of Survivin and Mcl-1 was calculated using the standard curve based method with GAPDH or 18s rRNA for normalization.

### Western blot analysis

Cells were lysed with RIPA buffer (Wako, Japan) or Laemmli’s sample buffer containing protease inhibitor cocktail (Nacalai tesque, Japan). After mixing, lysates were centrifuged at 15,000 rpm for 15 min at 4°C, and supernatants were separated. Western blot was carried out using standard procedures as described [50]. We performed Western blot at least three times for each target protein. Semi-quantification of the protein band intensities was done using the ImageQuant LAS4000mini (GE Healthcare, Japan).

### DNA sequencing analysis

Total RNA was isolated from MM cell lines using RNeasy Mini Kit (QIAGEN). cDNA was amplified by PCR using high-fidelity Taq DNA polymerase (PrimeSTAR HS DNA Polymerase, TAKARA BIO, Japan). The primers used to detect PSMB5 gene were: forward primer: 5′-CTTCAAGTTCCGCCATGGA-3′: and reverse primer: 5′-CCGTCTGGGAGGCAATGTAA-3′. After PCR amplification, the product was directly sequenced by the dye terminator method using ABI377 (Applied Biosystems, CA).

### Vector construction and retroviral infection

The DNA fragment coding for Survivin-GFP was obtained from pCMV6-Ac-GFP-Survivin (OriGene, Rockville, MD), and inserted into a retroviral vector pCX4bsr, a gift from Dr. Tsuyoshi Akagi (KAN Research Institute, Kobe, Japan). pBabe-Flag-Mcl-1 was obtained from Addgene. Vectors were packaged into viral particles by PEI-Max transfection of 8 μg of retroviral vectors along with 3.5 μg of pGP and 3.5 μg of pVSV-G (TaKaRa Bio/Clontech, Otsu, Japan) into HEK293T cells. U266 cells were infected with viral supernatant supplemented with 8 μg/ml polybrene at 37°C for 5 h, followed by overnight culture in virus-free growth media. The following day, the cells were subjected to a second round of infection, and then selected.

### Separation of primary MM cells from patients

Samples of human primary MM cells were obtained under Institutional Review Board approved protocols at our institution (University of Fukui). Residual bone marrow aspiration samples was obtained from patients with previously untreated MM. Mononuclear cells were separated using ficoll-paque solution (GE Healthcare). Then, the mononuclear cells were mixed with Cellbanker (Takara Bio Company, Japan) and stored in deep freezer. Each sample contained more than 80% of MM cells.

### Enrichment of G_0_/G_1_ cells

Cells enriched in the G_0_/G_1_ phase were obtained by incubating U266 in low serum medium (0.1% FBS) for 72 h and RPMI8226 in 0.2% FBS medium for 48 h.

### Cell cycle analysis by propidium iodide (PI)

Cells were washed with phosphate-buffered saline, fixed with 70% ethanol. Fixed cells were treated with RNase A (Sigma-Aldrich, MO), and stained with propidium iodide (PI). Samples were analyzed by BD FACS Canto II flow cytometer.

### Multiparameter flow cytometric analysis of G_0_ phase cells

This experiment was carried out according to a previous report [42]. U266 and RPMI8226 cells were incubated with the DNA dye Hoechst33342 (Sigma, St. Louis, MO) 10 μg/ml at 37°c for 45 min. The cells were washed with PBS, suspended in 1 ml PBS containing 0.1 μg/ml RNA-specific dye pyronin Y (Sigma, St. Louis, MO) at room temperature for 15 min. Flow cytometric analysis were conducted with a flow cytometer (BD FACSCanto II; Becton Dickinson, NJ). The population, which shows 2N DNA and lower levels of RNA is defined as G_0_ phase, the population which shows 2N DNA and normal levels of RNA is defined as G_1_ phase. 7-amino-actinomysin D (7-AAD) (Becton Dickinson, Franklin Lakes, NJ) staining (0.25 μg/ml at 37°c for 10 min) was used to detect dead cells.

### Silencing of survivin and Mcl-1 in MM cells

Small interfering RNA against survivin (Silencer Select siRNAs, assay ID; s1457), Mcl-1 (assay ID; s8583) and negative control siRNA were purchased from Applied biosystems (Foster City, CA). U266/BTZR1 cells were transfected with these siRNA by electroporation using Super Electroporator NEPA21 (NEPA GENE, Co. Ltd,. Ichikawa, Japan) according to the manufacturer's instructions.

### Xenograft murine model

NOD-SCID mice were purchased from Charles River Laboratories Japan, Inc. (Yokohama, Japan). Animal experiment protocol was approved by the institutional review board of International University of Health and Welfare, Tochigi, Japan. Cell suspensions in 100 μl of RPMI 1640 medium together with 100 μl of Matrigel basement membrane matrix (BD Biosciences) were inoculated subcutaneously into the flank of eight-week-old female NOD/SCID mice. Twenty days later, mice were randomly divided into two groups (n = 6/group). YM155 was administered intraperitoneally once daily at 5mg/kg for 9 days. Caliper measurements of the longest perpendicular tumor diameters were performed every alternate day to estimate tumor volume using the following formula: 4/3π × (width/2)^2^ × (length/2), which represents the three-dimensional volume of an ellipse.

### Statistical analysis

Statistical analysis was performed using two-tailed pared Student *t*-test on Excel software (ver. 14.5.7, Microsoft, WA). The following symbols were used: ^*^ and ^**^ that correspond to a *p* value inferior to 0.05 and 0.01, respectively.

## SUPPLEMENTARY MATERIALS FIGURES



## Abbreviations

MM: multiple myeloma
PBS: phosphate-buffered saline
CSC: cancer stem cell
IC_50_: the half maximal inhibitory concentration
