# Supplementary material for: YM155 exerts potent cytotoxic activity against quiescent (G0/G1) multiple myeloma and bortezomib resistant cells via inhibition of survivin and Mcl-1
Source: Oncotarget. 2017 Dec 4;8(67):111535–50. doi: 10.18632/oncotarget.22871 (PMC5762341; doi:10.18632/oncotarget.22871)
Supplement: Supplementary file 1 [file oncotarget-08-111535-s001.pdf]

YM155 exerts potent cytotoxic activity against quiescent (G_0/G_1) multiple myeloma and bortezomib resistant cells *via* inhibition of survivin and Mcl-1

SUPPLEMENTARY MATERIALS

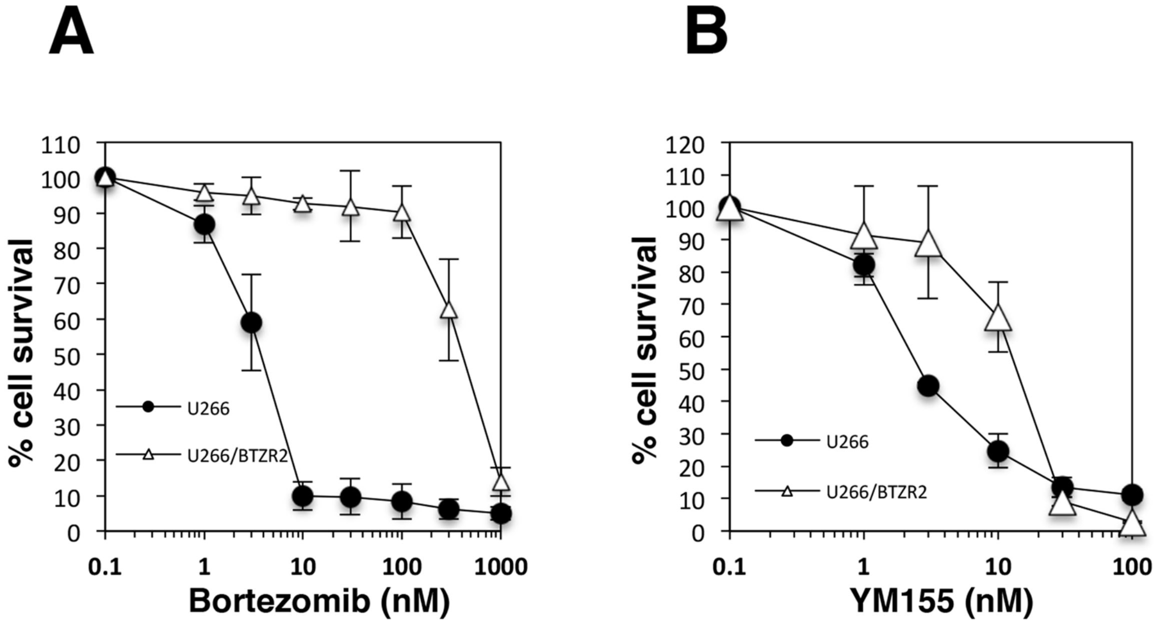

Supplementary Figure 1: Cell growth inhibition by YM155 in U266 or U266/BTZR2 cells. The cells were incubated with various concentrations of bortezomib **(A)** or YM155 **(B)** at 37°c for 72 h. Cell growth inhibition rate was determined by Cell counting Kit as described in Materials and Methods.

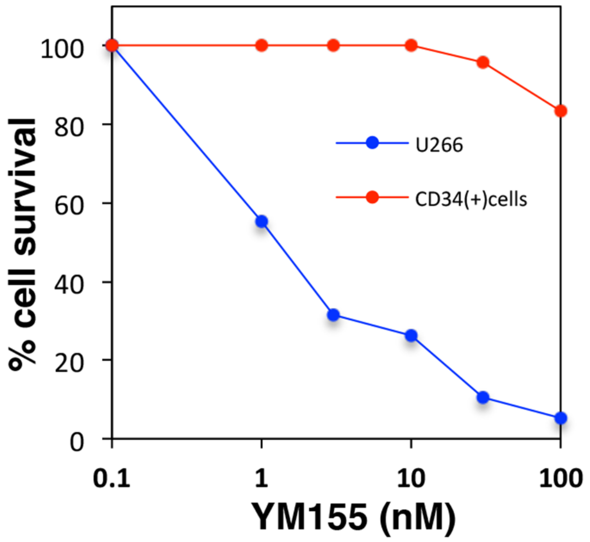

Supplementary Figure 2: Effect of YM155 on the growth of human normal CD34 (+) cells. CD34 (+) cells were incubated with various concentrations of YM155 at 37°c for 72 h. Cell growth inhibition rate was determined by trypan blue dye exclusion assay.
